# FITC-Dextran Release from Cell-Embedded Fibrin Hydrogels

**DOI:** 10.3390/biom11020337

**Published:** 2021-02-23

**Authors:** Viki Raz Lepsky, Sari Natan, Oren Tchaicheeyan, Avraham Kolel, Merav Zussman, Meital Zilberman, Ayelet Lesman

**Affiliations:** 1 Department of Materials Science and Engineering, Tel-Aviv University, Tel-Aviv, 55 Chaim Levanon St., Ramat Aviv 69978, Israel; vikilepsky@mail.tau.ac.il (V.R.L.); meravzussman@mail.tau.ac.il (M.Z.); meitalz@tauex.tau.ac.il (M.Z.); 2 School of Mechanical Engineering, Faculty of Engineering, Tel-Aviv University, Tel-Aviv, 55 Chaim Levanon St., Ramat Aviv 69978, Israel; sazor8@gmail.com (S.N.); toren101@gmail.com (O.T.); 3 Department of Biomedical Engineering, Tel-Aviv University, Tel-Aviv, 55 Chaim Levanon St., Ramat Aviv 69978, Israel; avrahamkolel@mail.tau.ac.il; 4 Center for Physics and Chemistry of Living Systems, Tel Aviv University, Tel-Aviv, 55 Chaim Levanon St., Ramat Aviv 69978, Israel

**Keywords:** drug delivery, controlled release, hydrogels, fibrin, FITC-dextran (FD), tissue engineering, extracellular matrix, traction force, cell-matrix interaction, regenerative medicine

## Abstract

Fibrin hydrogel is a central biological material in tissue engineering and drug delivery applications. As such, fibrin is typically combined with cells and biomolecules targeted to the regenerated tissue. Previous studies have analyzed the release of different molecules from fibrin hydrogels; however, the effect of embedded cells on the release profile has yet to be quantitatively explored. This study focused on the release of Fluorescein isothiocyanate (FITC)-dextran (FD) 250 kDa from fibrin hydrogels, populated with different concentrations of fibroblast or endothelial cells, during a 48-h observation period. The addition of cells to fibrin gels decreased the overall release by a small percentage (by 7–15% for fibroblasts and 6–8% for endothelial cells) relative to acellular gels. The release profile was shown to be modulated by various cellular activities, including gel degradation and physical obstruction to diffusion. Cell-generated forces and matrix deformation (i.e., densification and fiber alignment) were not found to significantly influence the release profiles. This knowledge is expected to improve fibrin integration in tissue engineering and drug delivery applications by enabling predictions and ways to modulate the release profiles of various biomolecules.

## 1. Introduction

Hydrogels, i.e., polymeric water-swollen and cross-linked networks [1], have been explored as attractive carriers for controlled drug delivery applications due to their biochemical and transport properties [2,3]. Such drug-eluting hydrogels are designed to maintain drug concentrations at effective levels over a prolonged period of time [4].

Specifically, acellular fibrin hydrogels have shown great potential in the controlled delivery of various pharmaceuticals [5,6,7,8]. Tredwell et al. [6] demonstrated the feasibility and effectiveness of the controlled release of cefazolin antibiotic over a 2-day period from fibrin sealant clots in vitro, with a sharp burst phase of release over the first 6–8 h, followed by a sustained release over the following 16 h. This system supports possible applications in orthopedic surgeries. Kara et al. [7] demonstrated that fibrin clots support the sustained and localized antibiotics release of vancomycin, ceftazidime, moxifloxacin and lomefloxacin, for postoperative ocular care. They demonstrated that the elution of all antibiotics decreased by 50% or more after 24 h. In addition, Jeon et al. [8] reported on the release of basic fibroblast growth factors from fibrin gels controlled by heparin and different concentrations of thrombin and fibrinogen. Thus, fibrin hydrogels can serve as an effective delivery system for various types of applications. In parallel, fibrin is also one of the primary biological scaffolds used to culture cells for tissue engineering [5,9,10,11,12,13], as it exhibits excellent biocompatibility, biodegradability, and tunable gel porosity and rigidity, while having the ability to be delivered in injectable or non-injectable forms to the damaged tissue [2,3,9,14]. However, although fibrin is often combined with both cells and therapeutic molecules, it remains unknown how the presence of cells affects drug release profiles.

As molecules must first diffuse to the hydrogel edge before they are released, diffusion measurements may provide insights and predictions into the effect of embedded cells on drug release profiles [2,15]. In this respect, Shkilnyy et al. [16] reported a ~17% reduction in the diffusion coefficient of rhodamine B when densities higher than 5 × 10^5^ of human umbilical vein endothelial cells (HUVECs) were integrated into fibrin gels. Suhaimi et al. [17] demonstrated that cell growth (human osteoblast HOSTE85) within scaffolds (collagen, PLLA and PCL) decreases the effective pore space of the scaffold, resulting in a decrease in the diffusion coefficient of glucose by about 13–26%. Kihara et al. [18] reported that the diffusion coefficients of biomolecules of different sizes (mainly 3–40 kDa dextran) decreased due to the remodeling of collagen fibers, and specifically fiber rearrangement and condensation by embedded cells. Leddy et al. [19] suggested that the decrease in the diffusivity of fluorescein isothiocyanate-dextran (FD) (3–500 kDa) in cellular constructs observed 28 days after cell seeding (by 27%) was due to new matrix synthesis by cells, while the increase in diffusivity (by 62%) observed over time in the acellular constructs was likely related to scaffold degradation and swelling. Overall, these diffusion studies indicate that the addition of cells to hydrogels typically results in a decrease in molecular diffusion, and this process is governed by various mechanisms such as pore size, fiber remodeling, matrix synthesis, matrix degradation, and swelling. However, the effect of incorporating cells to fibrin gels has yet to be quantitatively explored by means of molecular release profiles.

This work aimed to determine the impact of embedding cells (both of high and low contractility) in 3D fibrin hydrogels on the release of FD molecules. FD in the size of 250 KDa (radius of ~11 nm) was chosen as a model system of growth factors with comparable sizes. To this end, the release kinetics of FD from fibrin gels was monitored using a spectrophotometer for up to 48 h after the seeding of fibroblast or endothelial cells. Various contributing effects that can modulate the release rates were explored, including gel degradation by cells, cell-induced traction forces, cells acting as physical obstacles for diffusing molecules, and FD uptake by cells (adsorption and internalization) [20].

The results of this study provide predictions for the effect of embedded cells on the release profiles of therapeutic ingredients from fibrin hydrogels, as well as providing ways to modulate the release profiles, ultimately improving tissue regeneration efforts.

## 2. Materials and Methods

### 2.1. Cell Culture

NIH 3T3 fibroblasts (passages 33–53) stably expressing GFP-actin (obtained as gifts from Prof. S. Fraser, University of Southern California, Los Angeles, CA, USA) were cultured in DMEM medium supplemented with 10% fetal bovine serum (European Grade), 1% non-essential amino acids, 1% sodium pyruvate, 1% L-glutamine and 1% penicillin-streptomycin, in a 37 °C humid incubator. HUVECs (passages 4–7) were cultured in endothelial cell growth medium-low serum (2%), with SupplementMix, in a 37 °C humid incubator. All the materials were purchased from Biological Industries, Kibbutz Beit-Haemek, Israel.

### 2.2. Preparation of Fibrin Gels Embedded with Cells and FITC-Dextran

To create fibrin gels embedded with cells, 1 × 10^3^, 7 × 10^3^ or 15 × 10^3^ actin-GFP 3T3 fibroblasts or HUVECs (corresponding to 50 cells/µL, 350 cells/µL, and 750 cells/µL, respectively) were suspended in 10μL thrombin 10 U/mL (Omrix Biopharmaceuticals, Ness Ziona, IL, USA), included with 4.2 μg/μL FD 250 kDa (Sigma-Aldrich, Rehovot, Israel). Thereafter, 10 μL of the thrombin-FD-cell suspension was placed at the center of 24-well culture plates (Corning, NY, USA) and then mixed gently with 10 μL of 20 mg/mL human fibrinogen solution (Omrix Biopharmaceuticals, Israel). The resulting final fibrin gel concentration was 10 mg/mL fibrinogen and 5 U/mL thrombin, with a final FD concentration in the gel of 2.1 μg/μL. The resulting fibrin gel was placed in the incubator (37 °C, high humidity) for 45 min, for final polymerization, after which 1 mL warm cell growth medium was added to cover the gel. Gels without cells served as controls. All solutions were sterilized before use by filtering through 0.22 µm filters (Millex® GV, Darmstadt, Germany). In a part of the experiments, 10–14 μm polystyrene beads (SPHERO™, Spherotech, Inc., Lake Forest, IL, USA) were embedded in the fibrin gel (instead of cells) in order to examine their effect on the release profiles.

### 2.3. Determination of FITC-Dextran Cumulative Release from Fibrin Gels

**Figure 1:**
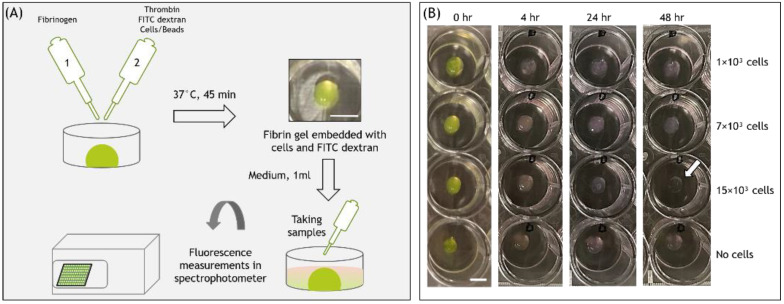
(**A**) Schematic of the experimental setup. (**B**) Images of fibrin gel 20μL embedded with FD 250 kDa and cells, at different time points from cell seeding. Scale bars are 500 μm.

At 1, 2, 3, 4, 24, and 48 h after cell seeding, two samples of 100 μL medium were collected from each 24-wells. The residual medium was completely removed, and replaced with 1 mL fresh medium. The FD content in a 100 μL sample was quantified using a fluorescence spectrophotometer (Synergy HT 2011, Bio-Tek Instruments, Winooski, VT, USA) at excitation and emission wavelengths of 485 nm and 528 nm, respectively (Figure 1). The spectrophotometer results of the two 100 μL duplicates (taken from the same well) were within a coefficient of variation (COV) lower than 8%. FD concentrations were calculated from calibration curves prepared using known concentrations. The cumulative release of FD was calculated using the following Equation (1):

(1) $$\%\mathrm{Cumulative}\;\mathrm{release}\;\mathrm{of}\;\mathrm{FD}\;=\;\frac{\mathrm{Mean}\;\mathrm{concentration}\;\left(\frac{\mu g}{\mu L}\right)\;\times \;\mathrm{Volume}\;\mathrm{of}\;\mathrm{the}\;\mathrm{medium}\;\mathrm{in}\;\mathrm{each}\;\mathrm{well}\;\left(\mu L\right)}{\mathrm{Total}\;\mathrm{amount}\;\mathrm{of}\;\mathrm{FITC}-\mathrm{dextran}\;\mathrm{in}\;\mathrm{the}\;\mathrm{gel}\;\left(\mu g\right)}\;\times \;100$$

As can be seen from Equation (1), cumulative FD release profiles were determined relative to the total amount of FD in the gel. The total amount equals the FD remaining in the gel at the end of the experiment (48 h) + the amount periodically measured in the medium during the incubation period (up to 48 h) [21]. Note that after 48 h, most of the FD was released from the fibrin hydrogel, i.e., more than 90%. To determine the amount remaining in the gel, the acellular gels incubated for 48 h were placed in 300 μL trypsin B solution and incubated at 37 °C until fully dissolved. The solution was then centrifuged (1.6 rpm, 7 min) and FD content in 100 μL of the supernatant was assessed using a fluorescence spectrophotometer. We noticed that the total amount (FD that released out of the gel plus the remaining in the gel) was typically 7% lower than the initial mass of FD added to each gel, measured manually using a scale. This difference was likely caused by the adsorption of FD to surfaces (tubes/dishes) with which it was in contact during the experiment. This 7% difference was identical for both cellular and acellular gels. Therefore, accumulated release calculations were adjusted by 7%.

### 2.4. Experiments with Dead Cells

Dead cells were prepared by placing the cells into a hot water bath at 65 °C for 30 min [22]. Then, the desired number of dead cells was added to the gel.

### 2.5. Treatment of Cells with Blebbistatin

After preparing the cellular gels, blebbistatin (a myosin II inhibitor, Sigma-Aldrich) [23,24] was added to the medium, to a final concentration of 83 μM. At every medium change (at 1, 2, 3, 4, 24, and 48 h), a fresh medium with the same concentration of blebbistatin was placed over the cells.

### 2.6. Autofluorescence Evaluation of Fibrin Degradation Products

1 × 10^3^, 7 × 10^3^, or 15 × 10^3^ cells were embedded in fibrin gels (with a final concentration of 10 mg/mL fibrinogen and 5 U/mL thrombin) without FD 250 kDa, and placed in the incubator (37 °C, high humidity), after which 1 mL warm cell growth medium was added to cover the gel. Forty-eight hours after cell seeding, the fluorescence intensity of the medium was examined.

### 2.7. Statistical Evaluation

**Figure 2:**
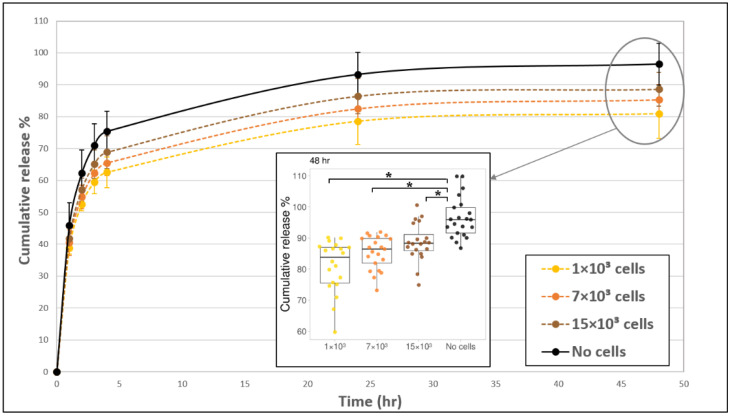
The addition of fibroblast cells to fibrin gels slowed down the release profile of FD 250 kDa. Fibroblast cells 1 × 10^3^ (yellow, dashed line), 7 × 10^3^ (orange, dashed line), and 15 × 10^3^ (brown, dashed line) were seeded fibrin gels and the release profile was monitored over time. Acellular gels (black, continuous line) served as the control. The values represent the mean ± SD of 20 gels. A statistically significant difference was observed between gels with 1 × 10^3^ cells versus without cells at all-time points, between gels with 7 × 10^3^ cells versus without cells during 2–48 h, between gels with 15 × 10^3^ cells versus without cells during 4–48 h, and between gels with 15 × 10^3^ cells versus 1 × 10^3^ cells during 2–24 h. Insert shows all data points at 48 h, for 20 gels of each tested condition. The boxplot shows the distribution of the experimental data with the range of variation, the interquartile range and the median. Significance with *p* < 0.05 is indicated by asterisks (*) in the boxplot.

All data were processed using the Statistical Package for the Social Sciences (SPSS) software (Version 25, IBM, Petah Tikva, Israel). Statistical comparison was evaluated by ANOVA and Bonferroni multiple pairwise tests. A normality test using the Shapiro–Wilk test was performed for all the experiments. Most of the results were found to be not normally distributed. Therefore, the one-way ANOVA on ranks (Kruskal–Wallis) test with Bonferroni post hoc analysis was used to explore statistical differences. For the results that were found to be normally distributed, we used the one-way ANOVA test with Bonferroni post hoc analysis. A value of *p* < 0.05 was considered statistically significant. Values provided are mean ± standard deviation (SD). PlotsOfData app [25] was used for statistical data visualization in Figure 2.

### 2.8. Fitting the Experimental Data with Mathematical Models of FD Release

The fit of the experimental data was performed according to the two-stage desorption theory [26,27], using a nonlinear least-square method in MATLAB^®^ R2020b, specifically by using fitnlm functionality. The confidence level was set as 95%. Each model function was evaluated by calculating and reporting a coefficient of determination (the R-squared measure of goodness of fit, R^2^) and root mean squared error (RMSE).

## 3. Results

### 3.1. Effect of Fibrin-Embedded Fibroblasts on FD Release Profile

**Figure 3:**
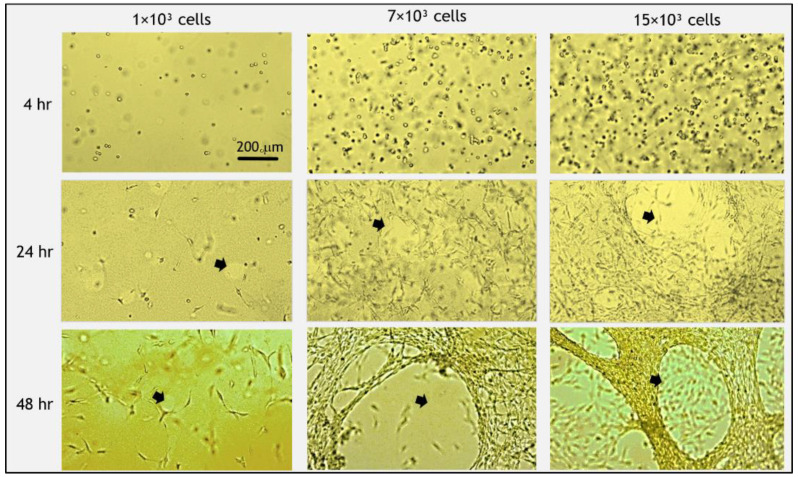
Morphology of fibroblast cells embedded in fibrin gels and gel degradation over time. Initially (<4 h post-seeding), cells were round and then spread out and elongated. At 48 h post-seeding, all cells took on a spread morphology. Degradation of the gel was apparent after 24 h, which accelerated at 48 h (arrows indicate holes of degradation). At higher cell concentrations, more gel degradation occurred. Scale bar is 200 µm.

The addition of fibroblast cells to the fibrin gel reduced FD release profile by about 7–15% (at 24–48 h), as compared to acellular gels (Figure 2). The exact magnitude of decrease was dependent on the amount of cells seeded in the gel. In general, the release profile from gels with 1 × 10^3^ cells was the lowest, and the release increased for the higher concentrations of 7 × 10^3^ and 15 × 10^3^ cells (but both were still lower than the acellular gels) with statistically significant differences between 1 × 10^3^ cells and 15 × 10^3^ cells during 2–24 h. The embedded fibroblast cells had a rounded morphology during the first 4 h post-seeding and began to spread out and take on an elongated morphology after approximately 5 h. The degradation of the gel by cells, manifesting as distinct holes in the gel, was apparent after 24 h (Figure 3, arrows). To assure that fibrin degradation products did not affect the spectrophotometer results by introducing autofluorescence, we measured the fluorescence signal from the medium of cell-degraded fibrin (without FD) and confirmed that there was no autofluorescence.

To better understand the mechanism of molecular release, we fitted a mathematical model to the experimental data of Figure 2. We found that the two-stage desorption theory [26,27] provides a good approximation (all R^2^ values > 0.995, and root mean squared error (RMSE) negligible (<1%)) for all evaluated models to a process that involves two characteristic time constants (Supplementary Information, Section I). In our case, one time constant (long sustained release, ~12 h) describes the apparent diffusion of FD in the gel, and another time constant (short burst, ~1 h) may describe repeated media flushing from a small volume gel to the outside container (gel/container volume ratio = 1/50); so we deal with nearly perfect sink conditions. Note that FD may also be transported from the gel surface during media change. The empty container is filled with fresh media each hour. Therefore, the actual drug release mechanism is a multi-stepped process where media are completely replaced each hour.

### 3.2. Effect of Fibroblast Cell Activities on FD Release Profile

**Figure 4:**
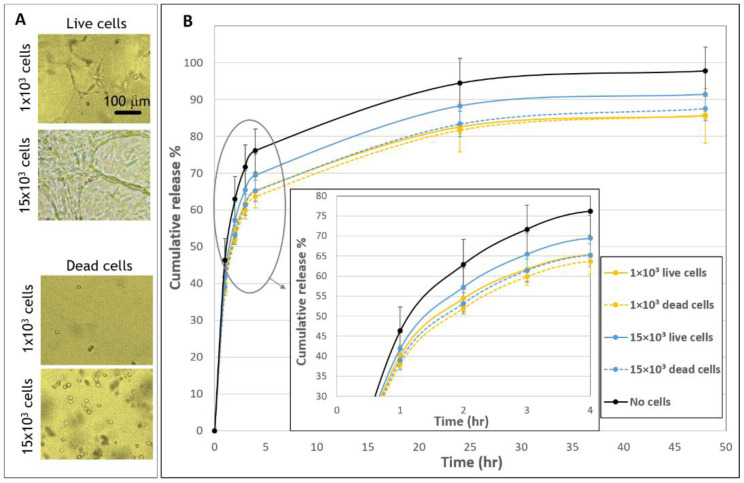
Release profiles from fibrin embedded with live versus dead fibroblast cells. (**A**) Morphologies of live and dead cells 48 h after seeded in fibrin gels. Scale bar is 100 µm. (**B**) The cumulative release of FD 250 kDa from live and dead cellular samples. Gels were embedded with 1 × 10^3^ live cells (yellow, continuous line), 1 × 10^3^ dead cells (yellow, dashed line), 15 × 10^3^ live cells (blue, continuous line), 15 × 10^3^ dead cells (blue, dashed line) or without cells (black, continuous line). A statistically significant difference in release profiles was noted between gels with cells (live/dead) compared to gels without cells. No statistically significant difference was found between gels with 1 × 10^3^ dead and live cells and between gels with 1 × 10^3^ and 15 × 10^3^ dead cells, for all time points. The values represent the mean ± SD of 30 gels for the live samples, 10 gels for the dead samples, and 29 acellular gels. Insert shows a zoom-in of the first hours after seeding (1–4 h).

The release profiles of molecules integrated into hydrogels can be affected by the activities of embedded cells, such as active cell-matrix interactions, including cell traction forces, gel degradation and the internalization of the diffusing molecules, as well as by passive effects, such as physical obstructions or the adsorption of diffusing molecules to cells’ surface. As a first attempt to gain more insight about the cellular-based mechanisms affecting release profiles, gels embedded with live versus dead cells were compared (Figure 4). Dead cells maintained a rounded morphology throughout the entire duration of the experiment, in contrast to the elongated shape of live cells (Figure 4A). The presence of dead cells reduced the release profile relative to acellular gels, in a manner that was similar to the effect of embedding live cells. This is indicated by significant differences in release profiles between cellular (both dead and live) and acellular gels (Figure 4B). Still, some difference was observed between live and dead samples in the high concentration of 15 × 10^3^ cells. Specifically, the decrease in the release profile was more notable for gels embedded with 15 × 10^3^ dead cells than with 15 × 10^3^ live cells. No significant difference was observed between the live and dead 1 × 10^3^ gels (Figure 4B).

**Figure 5:**
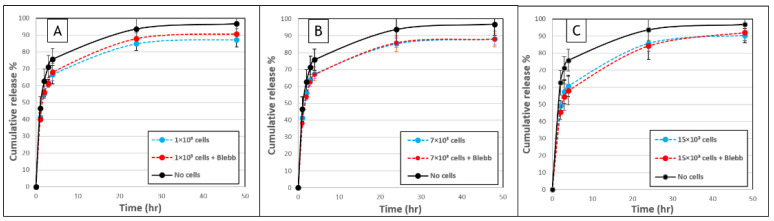
Fibroblast-induced forces do not modulate the release of FD 250 kDa from fibrin gels. (**A**) FD release from fibrin gels with (**A**) 1 × 10^3^ cells, (**B**) 7 × 10^3^ cells or (**C**) 15 × 10^3^ cells that were untreated (blue, dashed line), or treated with blebbistatin (red, dashed line), over a 48-h period. Untreated acellular gels served as controls (black, continuous line). No statistically significant difference was observed between cellular gels with versus without blebbistatin (*p* > 0.05). The values represent the mean ± SD of 8 gel replicates for each of the cellular gels and 21 gel replicates for the “no cells” gels.

To determine the role of cell-induced forces on the FD release profiles, gels were treated with blebbistatin, a well-established myosin II inhibitor, which arrests cell traction forces [23,24] (Figure 5). When comparing blebbistatin-treated to untreated cell-embedded gels, similar profiles of release were recorded, suggesting that cell-generated forces do not significantly modulate the release profiles during 48 h post-seeding interval.

FD uptake by cells (internalization or absorption) could impact the release profiles. Therefore, to examine this possible effect, the change in FD in the medium around cells cultured on plastic wells in comparison to wells without cells was examined after 48 h (Supplementary Information, Section II). No statistically significant difference was found in the fluorescence intensity of FD in wells with 1 × 10^3^ cells, 7 × 10^3^ or 15 × 10^3^, relative to wells without cells (*p* > 0.05), indicating negligible uptake of FD 250 KDa by cells.

### 3.3. Effect of Fibrin-Embedded HUVECs on the FD Release Profile

**Figure 6:**
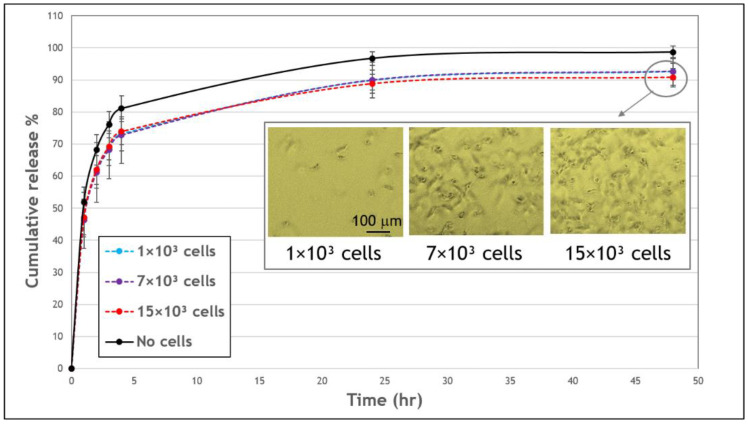
The effect of human umbilical vein endothelial cells (HUVECs) on the cumulative release profile of FD 250 kDa from fibrin gels. Gels embedded with 1 × 10^3^ (blue, dashed line), 7 × 10^3^ (purple, dashed line), 15 × 10^3^ cells (red, dashed line), or without cells (black, continuous line). No statistically significant difference was noted in FD release profiles from gels with compared to without cells over the 48-h observation period (*p* < 0.05). The values represent the mean ± SD of 8 gels. Insert shows images of HUVECs embedded in fibrin gels, after a 48-h incubation. Scale bar is 100 μm.

The effect of populating the fibrin gels with endothelial cells was also assessed. Endothelial cells are considered to have a reduced contractile activity and interaction with the surrounding matrix in comparison to fibroblasts; therefore, these two cell types were investigated and compared. HUVECs seeded in fibrin gels loaded with FD 250 kDa (Figure 6) led to at most 6–8% reduction in FD release profiles, as compared to its release from acellular gels, with no statistically significant difference between gels with compared to without cells over the 48-h observation period. Additionally, in the case of HUVECs and in contrast to fibroblasts, the release profiles were similar for all tested endothelial cell densities, and throughout the tested period. In addition, no gel degradation was apparent over the 48-h observation period.

### 3.4. Effect of Polystyrene Beads on the FD Release Profile

**Figure 7:**
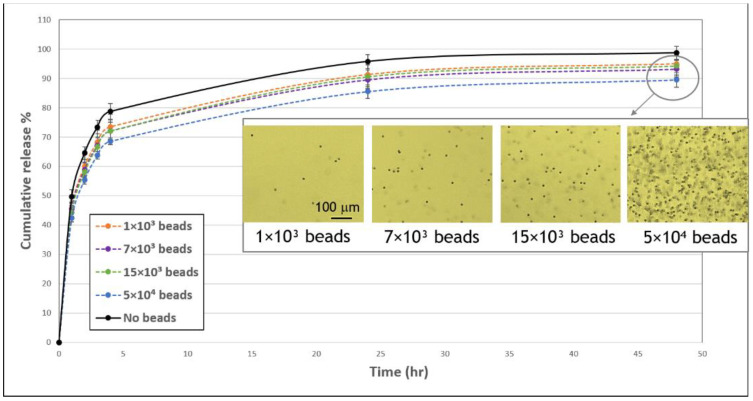
Influence of beads on the cumulative release profile of FD 250 kDa from fibrin gels. FD-loaded gels were embedded with 1 × 10^3^ (orange, dashed line), 7 × 10^3^ (purple, dashed line), 15 × 10^3^ (green, dashed line), or 5 × 10^4^ (blue, dashed line) or without (black, continuous line) polystyrene beads (10.2 μm) and FD release was monitored over the subsequent 48-h period. A statistically significant difference in FD release from gels with 7 × 10^3^, 15 × 10^3^, and 5 × 10^4^ versus without beads was noted throughout the observation period (*p* < 0.05). The values represent the mean ± SD. The number of replicates is N = 9, 11, 10, 11 and 5 for: no beads, 1 × 10^3^, 7 × 10^3^, and 15 × 10^3^, 5 × 10^4^ beads, respectively. Insert shows images of bead-embedded gels after 48 h. Scale bar is 100 μm.

To gain further insights into the mechanisms affecting release profiles, we next examined the effect of embedding polystyrene beads of comparable size to cells (~ 10 µm) on FD release. To mimic the cell system, 1 × 10^3^, 7 × 10^3^, or 15 × 10^3^ beads were embedded in each gel, corresponding to volume fractions of 0.04%, 0.25%, and 0.54% (*v*/*v*). A higher concentration of 5 × 10^4^ beads, corresponding to a volume fraction of 1.81% (*v*/*v*), was also tested. The presence of beads decreased the release profile of FD by about ~5–6% compared to gels with no beads, with a statistically significant difference. No significant differences were noted between the three lowest tested quantities of beads (Figure 7). In contrast, the highest tested concentration of beads (5 × 10^4^) reduced the release profile more significantly by ~9–10%.

## 4. Discussion

Fibrin gels are commonly used as 3D culture models to study and simulate physiological environments for the exploration of cell-matrix interactions [28]. Fibrin gels are also routinely used as scaffolds for tissue engineering to support cellular activities such as growth, differentiation, and proliferation. Fibrin is also a central polymer carrier in drug delivery applications. Many research efforts were dedicated to understanding the release of molecules/drugs from fibrin gels. Specifically, it was shown that various parameters affect the release, including the density and pore size of the hydrogel, formulation, e.g., the presence of heparin [8,29,30], the molecular weight of the released substance [31], and tissue/organ interfaces that may apply external forces (i.e., compressive, tensile, and shear forces) on the gel [32]. Yet, although fibrin gels are commonly combined with biomolecules and cells in these various applications, the effect of embedded cells on release profiles has yet to be quantitatively determined.

Indirect insights into the effect of cells on drug release are currently available only from diffusion studies that measured the diffusion coefficient of different molecules in the gel with and without cells. In this study, in contrast, we were interested in understanding the flow of molecules/drugs directly out of the gel, and the influence of the embedded fibroblasts and endothelial cells in fibrin gels on the release profile of FD 250 kDa. FD was selected as a model due to its wide range of molecular weights, high stability, and frequent use in drug release studies and diffusion analysis [19,33,34]. FD 250 kDa has an approximate radius of 11 nm, which is comparable to the size of growth factors, such as nerve growth factor (130 kDa, globular radius ~3 nm, unfolded ~12 nm) and hepatocyte growth factor (80 kDa, globular radius ~3 nm, unfolded ~9 nm) [35,36,37]. Thus, the specific size of FD used in this study can provide an interesting and simple initial prediction for these growth factor release profiles.

In our system, we focused on low-volume gel discoids (20 μL, lens-shaped) that are fixated (adherent) to a flat substrate (a plastic dish). Therefore, the gel was partially mechanically constrained and minimal deformation occurred along the x-y plane (substrate plane). No constraint was applied on the thickness of the gel, so some shrinkage occurred along the z-axis (its height). We used this specific system in order to simulate the layering of gels on a targeted tissue in the body. We emphasize that the gel discoids’ shape was plano-convex (similar to a truncated spheroid or spread-drop-like) and did not float freely as spheres within the wells. The choice of 20 μL fibrin volume was intended to simulate the delivery of molecules into relatively small areas of tissues, as was performed by Nunes e Silva et al. [38] in their clinical research for delivering nerve growth factor with fibrin glue into peripheral nerves, or by Galler et al., who injected 20 μL of cells-embedded (Polyethylene glycol) PEGylated fibrin hydrogel into small dental defects in animal studies [39].

The composition of the fibrin gel used in this study (10 mg/mL fibrinogen) was chosen to allow suitable gel conditions to embed cells in 3D and support their growth, migration, and proliferation, as shown by us [40] and others [16]. The concentration of cells was chosen in the range of 1–15 × 10^3^ (50–750 cells/µL) due to the fact that using lower concentrations will increase the dispersion of the cells, which may affect their growth by impeding cell–cell communications [41,42]. On the other hand, from our experience with these fibroblast-embedded fibrin gels, using a higher cell concentration in the current formulation will cause too rapid degradation of the gel [43,44,45], which we wanted to avoid. Furthermore, a similar cell concentration was used in various related previous studies. Kim et al. [46] evaluated the biocompatibility of drug-loaded fibrin gel using approximately 200 cells/µL of human mesenchymal stem cells and found this formulation to be viable for cells in terms of increased proliferation and osteogenic differentiation. In addition, van Esterik et al. [43] and Jansen et al. [47] demonstrated the effect of cell-generated forces on the properties of the fibrin network using an approximate concentration of 500 fibroblasts cells/μL. Moreover, Ye et al. [48] estimated the biodegradability and the potential of 3D fibrin gels seeded with 750 cells/μL human myofibroblasts in cardiovascular tissue engineering and found it suitable for tissue development.

It is widely known that cells have the capability to exert traction forces (e.g., fibroblasts) and often contract the surrounding gel matrix, locally and globally. Our initial assumption that had motivated this research was that cellular traction forces in fibrin gels will cause higher release profiles of drugs compared to the release from acellular gels, as forces are expected to cause gel global shrinkage [49] and induce the extraction or expulsion of liquid from the gel (i.e., syneresis) [50,51,52]. During active syneresis, we expected advection to occur, i.e., directed transport of molecules/drugs out of the gel and therefore faster and higher release profiles. A related example is the activated platelets-fibrin gel system in which the developed contractile stress of a blood clot may impact the release of different molecules from fibrin gels [53,54,55,56].

The influence of acto-myosin cellular forces on release profiles was examined by analyzing release profiles in the presence of blebbistatin, a myosin II inhibitor. Overall, gel-embedded cells treated with blebbistatin had restricted movement of their protrusions and their cell body with reduced interaction with the surrounding fibrous gel, apparent by a significant decrease in gel densification around treated cells, as we showed in our previous study [57]. Cellular forces were found to have a negligible effect on the release profile of FD 250 kDa from the fibrin gels used in this study (10 mg/mL fibrinogen and 5 U/mL thrombin). We suggest that this could be due to the weak cellular traction forces versus the relatively strong attachment of the gel to the substrate (boundary constraints on gel shape deformation), and the fast FD molecule diffusion relative to slow deformation and degradation rate. Thus, the results of this work demonstrate that other mechanisms are involved, as will be discussed below in more detail. 

In our system, we examined the influence of embedded fibroblast or endothelial cells on FD release profiles (Figure 2 and Figure 6). Fibroblast cells are known to interact more substantially with the extracellular matrix, applying larger traction forces as compared to endothelial cells [58]. Fibroblasts also degrade the fibrin matrix more extensively than endothelial cells, as we observed in our experiments (Figure 2 and Figure 5). In addition, endothelial cells and fibroblasts are known as important constituents that are naturally entrapped in fibrin clots during the blood clotting process [3]. Therefore, the effect of these two cell types was investigated and compared. The addition of fibroblast cells to fibrin gels decreased the release profile of FD by approximately 7–15% (value at 48 h) in comparison to release from acellular gels (Figure 2). However, interestingly, higher fibroblast concentrations typically enhanced FD release. A similar situation was observed in a study by Shkilnyy et al. [16], who reported a ~17% decrease in the diffusion rate of rhodamine B when cells were included in the same fibrin gels; however, the diffusion rate was increased when cell concentrations were relatively elevated, yet still lower than the diffusion rate in acellular gels. This effect can be ascribed to the combined influence of multiple concurrent processes, both active and passive ones. For example, at higher cell concentrations, gel degradation may enhance the release of the reporter molecule; however, at the same time, more cells can also physically obstruct diffusing molecules or uptake FD, thereby impeding their release. In the case of fibroblast cells, degradation holes caused by tension [45] were indeed observed in the gels, which may likely increase FD release at incubation times of >24 h. The uptake of FD 250 KDa molecules by fibroblast cells was found to be negligible in our system, which is also in line with a previous study that showed a significant decrease in internalization with the size of the FD molecules, with a possible uptake by cells up to FD 150 kDa [20]. We note that the measurement of FD uptake by cells was performed in a 2D fibroblast cell culture, an environment different from 3D gel conditions. The 2D system was chosen here as it provided a simpler and more direct system to measure cell uptake, without involving side effects contributed by the presence of a gel, such as its degradation by cells. A somewhat different situation was observed for endothelial cells, where almost no gel degradation was apparent, and indeed, the release profile was less affected by the number of cells seeded in the gel. These findings align with those of molecular diffusion studies [16,17,19], where a decrease in molecular diffusivity was measured in cellular as compared to acellular constructs.

Two types of experiments were conducted in an attempt to isolate the passive from the active cell activities. First, we compared the effect of live versus dead cells (Figure 4). We used a simple heat method [22], which allowed us to use dead cells immediately after their exposure to heat without the intervention of other materials (e.g., organic solvents, chemical crosslinkers, poisons) that may impact the experimental system. At both concentrations of dead cells (1 × 10^3^ and 15 × 10^3^), we observed practically the same release profile, and both lowered the release profile relative to acellular gels, implying that passive mechanisms are at work but have low concentration dependence. Second, the release profiles of FD gels embedded with polystyrene beads were analyzed, allowing us to better understand the effect of physical obstruction to diffusion (Figure 7). This is under the assumption that FD internalization is lessened in the case of beads, although some adsorption between these beads and the FD may still occur. When embedding beads of sizes comparable to those of cells, release profiles were decreased, indicating the effect of cell-physical interference on molecular diffusion and, ultimately, on release profiles. It is surprising that this physical interference effect was already evident at very small volume fractions of beads/cells (0.004%, 0.03%, 0.07%, and 0.2% *v*/*v*). Similarly, Shkilnyy et al. [16] reported that a cell volume fraction as small as 0.21% (*v*/*v*) was sufficient to reduce rhodamine B diffusion. Beyond basic understanding of the mechanisms regulating release profiles, adding beads to gels establishes a potential simple system to modulate drug delivery for applications in regenerative medicine.

The addition of different molecules to the cells-embedded hydrogel, such as growth factors and drugs, can improve tissue regeneration and the function of the engineered tissue in the body. Our in vitro results can contribute to the prediction of drug release from hydrogels populated with cells. However, many challenges have to be considered during the development of a new tissue. Therefore, additional experiments that simulate the effects of relevant body mechanisms should be performed before the stage of in vivo studies, for example, the impact of external forces [32] (i.e., compressive, tensile, and shear forces) and other environmental conditions in the body (including pH, enzymes, and temperature) on molecular release from cells-embedded hydrogel, and also to optimally formulate the selected gel concentrations (optimal gel mechanical properties) [32]. In addition, one should assure that a high drug concentration within the cell-embedded gel does not interfere with normal cell physiology by direct or indirect toxicity. Moreover, it will be interesting to further explore the extent of syneresis caused by cell-driven endogenous traction forces, for example, using freely floating spherical gels where embedded cells may experience low-resistance isotonic-like contraction; in contrast to conditions experienced by the cells in our system, where the gels are attached to a rigid surface on one side and cells may experience this mechanical boundary constraint, and therefore isometric-like contraction occurs. In that case, the release profile of reporter molecules/particles with a characteristic diffusion time longer (e.g., higher molecular weight of FD than the 250 kDa) than the characteristic time of cell-induced gel contraction should be studied; similarly, it would be interesting to see how these profiles are affected by cells with stronger single-cell traction force (e.g., comparing cardiomyocyte versus fibroblast induced release). The motivation here is to learn how gel/cells’ mechanical conditions affect FD release; this, in turn, may provide new insights about the effect of cell traction-forces on the release profile of drug molecules eluting from the gels and the role of flow through the gel pores to the outside.

## 5. Conclusions

In this study, we show that cells embedded in fibrin gels demonstrate mildly decreased release profiles of 250 kDa FD over a 48-h period compared to acellular gels. This modulatory effect was primarily imparted by the active gel degradation by cells and the passive presence of cells as obstacles for molecule diffusion. This knowledge can contribute to the prediction of drug release from hydrogels populated with cells, a common element of tissue engineering applications. It can also offer a system to manipulate drug delivery by populating gels with synthetic beads at controllable volume fractions. Future research, such as in vivo experiments, will be essential to establish the applicative potential of these findings.

## Data Availability

Data is contained within the article or supplementary material.

## Supplementary Materials

The following are available online at https://www.mdpi.com/2218-273X/11/2/337/s1.
