# Supplementary material for: FITC-Dextran Release from Cell-Embedded Fibrin Hydrogels
Source: Biomolecules. 2021 Feb 23;11(2):337. doi: 10.3390/biom11020337 (PMC7926394; doi:10.3390/biom11020337)
Supplement: Supplementary file 1 [file biomolecules-11-00337-s001.pdf]

<u>Section I-</u> Mathematical Model Fit of Experimental Data:

The fit of the experimental data was performed according to the two-stage desorption theory [1-2], using a nonlinear least-Squares method in MATLAB® R2020b, specifically by using ***fitnlm*** functionality. The confidence level was set as 95%. Each model function was evaluated by calculating and reporting a coefficient of determination (the R-squared measure of goodness of fit, R^2) and root mean squared error (RMSE). All R^2 values were found very close to one, and RMSE was found negligible for all evaluated models (typically < 1%). Residuals were evenly distributed.

The two-stage desorption theory is a good approximation to a process that involves two characteristic time constants. In our case, one time constant (long, ~12 Hr) describes apparent diffusion of FD in the gel, and another time constant (short, ~1 Hr) may describe repeated media flushing from a small volume gel to the outside container (gel/container volume ratio = 1/50) ; so we deal with nearly perfect sink conditions. Note that FD may also be transported from the gel surface during media change. The empty container is filled with fresh media each hour. Therefore, the actual drug release mechanism is a multi-stepped process where media is completely replaced each hour. Of course, these time constants have some variation and affected by many parameters such as batch-to-batch difference in gel consistency, gel being with or w/o embedded beads or cells, gel shape (surface to volume ratio) and media to gel volume ratio.

A mathematical model that closely approximates a two-stage process can be described by the following equation[1,2]:

$$(1)\quad \frac{M_t}{M_{d0}} = b_1 \cdot \left[1 - exp\left(-\frac{t}{b_2}\right)\right] + b_3 \cdot \left[1 - exp\left(-\frac{t}{b_4}\right)\right]$$

Where M_t is the amount of released FITC-dextran over time t, M_{d0} is the total initial amount of FITC-dextran in the fibrin gel. $b_1\&b_3$ and $b_2\&b_4$ are related to the amount and rate of the release process, respectively. $b_1\&b_2$ correspond to drug release from the fast acting process, and $b_3\&b_4$ correspond to the slowly leachable component release. The amount released at t=∞ is $R_\infty = b_1 + b_3$. Ideally, R_∞ should be 1, but in practice it is smaller due to the loss of FD to adhering gel fibers/ membranes/ vessel walls.

In the above-described model one needs to fit four parameters (b_1, b_2, b_3 and b_4). We wanted to check how good would be the fit if we use the information that one-time constant is close to 1 hour (repeated media replacement in the well). So one may fixate the parameter $b_2 = 1$:

(2) $\frac{M_t}{M_{d0}} = b_1 \cdot [1 - exp(-t)] + b_3 \cdot \left[1 - exp\left(-\frac{t}{b_4}\right)\right]$

In the above-described model one needs to fit three parameters (b_1, b_3 *and* b_4). We noticed from the results of a non-linear fit (see below) that most of the FD is released during the first few hours and only a relatively lower amount is released during the following hours. The parameters $\frac{b_1}{R_\infty} \approx \frac{2}{3}$ & $\frac{b_2}{R_\infty} \approx \frac{1}{3}$. We wondered what would be the effect of fixating the values of these parameters ($\frac{b_1}{R_\infty} = \frac{2}{3}$; $\frac{b_2}{R_\infty} = \frac{1}{3}$) on the goodness of fit. Degeneration of the model from four to a two variables (R_∞ and b_4) effect on goodness of fit was explored:

(3) $\frac{M_t}{M_{d0}} = R_\infty \cdot \left\{\frac{2}{3} \cdot [1 - exp(-t)] + \frac{1}{3} \cdot \left[1 - exp\left(-\frac{t}{b_4}\right)\right]\right\}$

Our conclusion regarding fitting procedure is that the two-stage desorption theory mathematical expression is a good approximation to the actual behavior of FD 250 KDa release from fibrin gels as described in this paper; we demonstrate excellent goodness of fit values, even for a degenerated two parameters fitted expression. The four parameter expression is the model of choice, however a practical, and simpler description may be obtained by a two parameter fit expression that describes a simple first order process convolved with a multi-stepped media refreshment process. Below we show the fitting results for acellular gels and gels with various amounts of fibroblast cells, but we note that the fitting to all other conditions reported in the manuscript (dead cells, beads, blebbistatin) had R^2 values > 0.995, and RMSE was found negligible for all evaluated models (typically < 1%), indicating excellent fit.

Table 1. Parameters obtained by fitting of Eq.1, 2 & 3 the to the experimental data for FITC-dextran Release from fibrin gels with fibroblast cells (Figure 2).

				R_∞ = 99.076
**No cells**	b	Eq.1	Eq.2	Eq.3
	b_1 [%]	68.194	68.495	66.051
	b_2 [hour]	0.992	*	*
	b_3 [%]	31.328	31.086	33.025
	b_4 [hour]	10.745	10.939	8.715
	R^2	1	1	1
	RMSE [%]	0.672	0.584	0.767
				R_∞ =83.997
**1000 cells**	b	Eq.1	Eq.2	Eq.3
	b_1	55.768	57.751	55.998
	b_2	0.938	*	*

	b_3	28.167	26.537	27.999
	b_4	9.934	11.316	9.404
	R^2	1	1	1
	RMSE [%]	0.316	0.448	0.618
				R_∞ =88.465
**7000 cells**	b	Eq.1	Eq.2	Eq.3
	b_1	58.612	60.303	58.977
	b_2	0.948	*	*
	b_3	29.724	28.360	29.488
	b_4	10.470	11.639	10.212
	R^2	1	1	1
	RMSE [%]	0.436	0.494	0.587
				R_∞ = 91.608
15000 cells	b	Eq.1	Eq.2	Eq.3
	b_1	60.105	61.763	61.072
	b_2	0.959	*	*
	b_3	31.355	29.939	30.536
	b_4	8.899	9.800	9.205
	R^2	1	1	1
	RMSE [%]	0.420	0.423	0.413

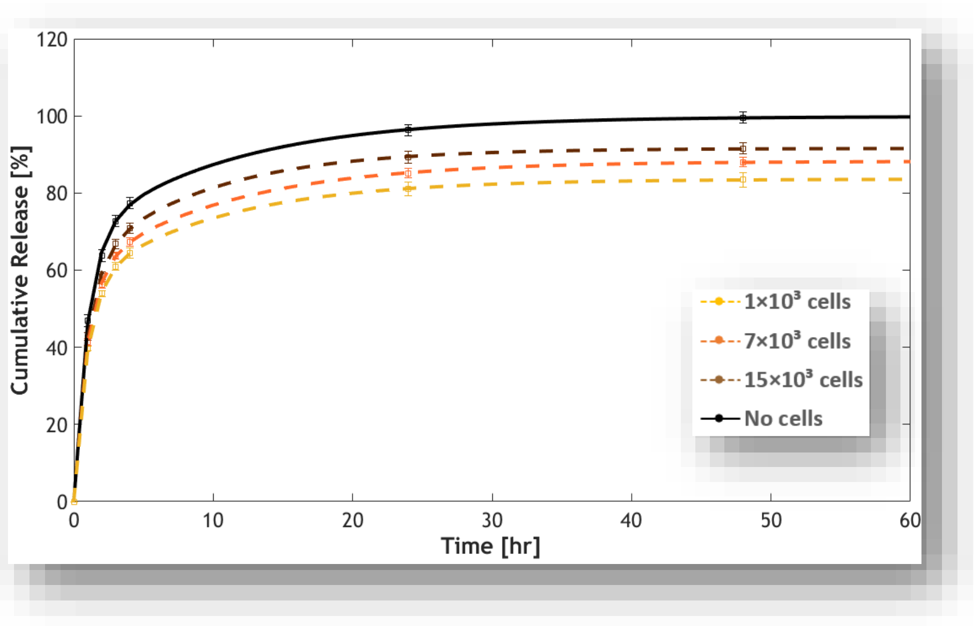

Figure S1: Mathematical model fitting (lines) to experiential data (points) described in Figure 2.

Bibliography:

1. Khansari, S. *et al.* Two-Stage Desorption-Controlled Release of Fluorescent Dye and Vitamin from Solution-Blown and Electrospun Nanofiber Mats Containing Porogens. *Mol. Pharm.* **10**, 4509–4526 (2013).

2. Zupančič, Š., Sinha-Ray, S., Sinha-Ray, S., Kristl, J. & Yarin, A. L. Long-Term Sustained Ciprofloxacin Release from PMMA and Hydrophilic Polymer Blended Nanofibers. *Mol. Pharm.* **13**, 295–305 (2016).

Section II- Evaluation of FD 250 kDa uptake by cells

To test the possibility that cells uptake (internalize/adsorb) the FD 250 kDa molecules, 1×10^3, 7×10^3, or 15×10^3 actin-GFP 3T3 fibroblasts cells were seeded on 96-well culture plates (Corning, US) with 200 µl of FD 250 kDa 2.1 µg/µl in their surrounding medium and incubated (37 °C, 5% CO_2, high humidity) for 48 hours. 7 wells/repetitions were used for each cell quantity. To determine the fluorescence intensity of FD 250 kDa after 48 hours at each plate, a sample of 100 µl was collected from each well-plate and quantified using a fluorescence spectrophotometer (Bio-Tek Instruments, Synergy HT_2011) at excitation and emission wavelengths of 485 nm and 528 nm, respectively. No statistically significant difference in the fluorescence intensity of FD between wells with 1×10^3 cells, 7×10^3 or 15×10^3, and wells without cells ($p>0.05$), indicates negligible internalization by cells.

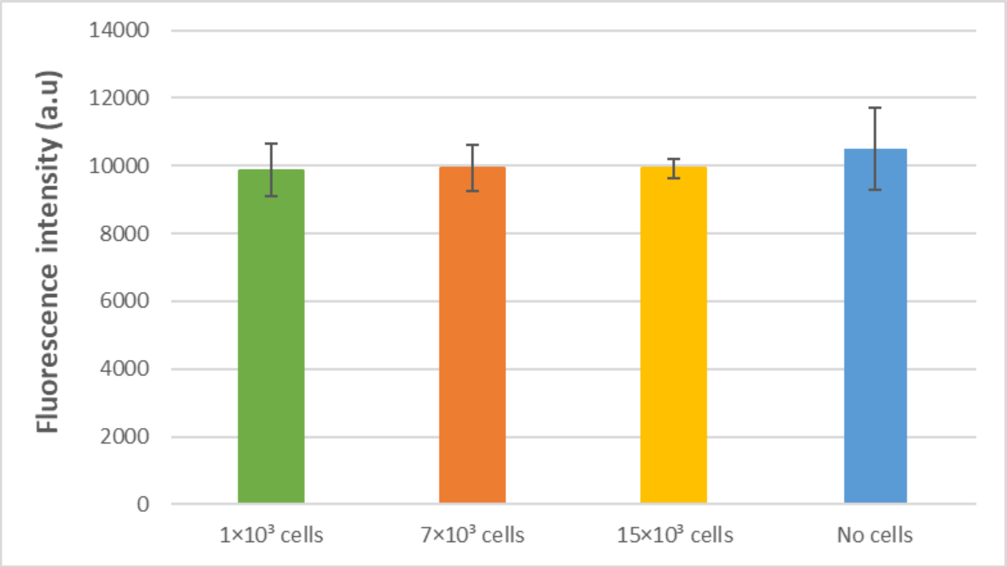

Figure S2: FD 250 kDa internalization by 1×10^3, 7×10^3, and 15×10^3 fibroblast cells seeded on 96 wells, after 48 hours. No statistically significant difference was found between 1×10^3 cells, 7×10^3 or 15×10^3, in comparison to no-cells ($p>0.05$), indicating negligible internalization by cells ($p>0.05$). n = 7 wells/repetitions for each cell quantity.
